# The Association Between Total Percent Fat and Serum Uric Acid in Adults

**DOI:** 10.3389/fnut.2022.851280

**Published:** 2022-05-20

**Authors:** Juan Sun, Chenyang Yue, Zhen Liu, Jie Li, Weiming Kang

**Affiliations:** ^1^Division of General Surgery, Department of Surgery, Peking Union Medical College Hospital, Peking Union Medical College, Chinese Academy of Medical Sciences, Beijing, China; ^2^Department of Biology, York University, Toronto, ON, Canada

**Keywords:** total percent fat, serum uric acid, obesity, hyperuricemia, NHANES

## Abstract

**Background:**

Serum uric acid (SUA) has been proven to be closely associated with metabolic abnormalities, including obesity. This study aimed to investigate the detailed relationship between total percent fat (TPF) and SUA among adults.

**Methods:**

Briefly, 23,715 adults aged 18–59 years in the National Health and Nutrition Examination Survey (NHANES) 1999–2018 were included in this study. Multivariable linear regression models were used to examine the association between TPF and SUA. Subgroup analyses stratified by sex and obesity status were also performed by multivariable linear regression. Then, fitted smoothing curves and generalized additive models were also applied to address the non-linear relationship between TPF and SUA. Finally, a recursive algorithm was used to calculate the inflection point in the non-linear relationship and a two-segment piecewise linear regression model was used to analyze the relationship between TPF and SUA on both sides of the inflection point.

**Results:**

There were 15,808 (66.7%) obese individuals in all 23,715 participants. In the fully adjusted model, there was a positive association between TPF and SUA (β = 0.99, 95% CI: 0.73–1.26). Besides, this positive association remained statistically significant in subgroup analyses stratified by sex and obesity status. Interestingly, in males, the association of TPF and SUA was an inverted U-shaped curve (inflection point: 34.3%).

**Conclusion:**

Our study revealed a significant positive relationship between TPF and SUA among adults and this association remained statistically significant when stratified by sex and obesity status, but the shape of the smoothing curve in males differed from that in females.

## Introduction

Obesity has gradually become a major public health concern owing to its increased risk of various diseases. The negative impacts on quality of life and overall life expectancy in the obese populations, as well as the extra healthcare expenditures on obesity-related comorbidities, emphasize the need for a more detailed clinical assessment to allow effective obesity management (1, 2). Characterized by an increase in the number and/or the size of fat cells and the unusual distribution of fat mass, obesity cannot be accurately measured by body mass index (BMI) because it has difficulty differentiating lean mass from fat mass, and peripheral fat from central fat. This limitation makes BMI unable to identify normal weight obesity (NWO), a phenomenon associated with increased cardiovascular mortality and a high risk of developing metabolic abnormalities, making BMI an imprecise indicator of these negative events (3, 4). Compared with other techniques, dual-energy X-ray absorptiometry (DXA) not only evaluates body fat mass and its distribution with high precision, but also distinguishes fat mass from bone mass and lean mass (5). Notably, visceral adipose tissue measured by DXA has been confirmed to be associated with metabolic characteristics (6–8). In addition, the minimal radiation exposure and strong clinical feasibility also make DXA the preferred method for body composition assessment (5).

Hyperuricemia (HUA), defined as serum uric acid (SUA) levels >415 μmol/L in males and >340 μmol/L in females (9), is associated with a wide range of health outcomes, such as cardiovascular diseases, metabolic syndrome, and cancer (10). The prevalence of HUA is increasing worldwide, affecting approximately one out of five adults in the United States (11). A large-scale longitudinal study found that obese patients had a higher risk of developing HUA (12). Though many other studies indicated that SUA was closely related to obesity (13–15), the obesity mentioned in these studies were mainly measured by BMI. Since it was shown that SUA was more significantly correlated with fat mass (16, 17), the relationship between TPF and SUA seems to be much more meaningful.

The purpose of this study is to evaluate the detailed relationship between TPF and SUA using a large nationally representative sample of US adults. Furthermore, due to the male-female differences in TPF and SUA, subgroup analyses were carried out following the STROBE guidelines (18) to illustrate the associations in different subgroups.

## Materials and Methods

### Study Population

Data were from the 1999–2018 National Health and Nutrition Examination Survey (NHANES), a continuous surveillance survey to assess the health and nutritional status of a representative sample of the United States civilians. Data of 40,117 participants aged 18–59 years were retrieved from the database. After excluding participants with incomplete TPF and SUA data (*n* = 16,402), participants with complete TPF and SUA data were included in our subsequent analyses (*n* = 23,715). The NHANES was reviewed and approved by the National Centre for Health Statistics Research Ethics Review Board and the informed consents were obtained from all participants in each year’s survey.

### Variables

The exposure variable, TPF, was measured by whole body DXA scan using QDR-4500 Hologic Scanner (Bedford, MA, United States). The outcome variable, SUA, was measured by 704 Multichannel Analyzer or Roche Hitachi Model 917 from 1999 to 2001, Beckman Synchron LX20 from 2002 to 2007, Beckman Coulter UniCel^®^ DxC800 from 2008 to 2016, and Roche Cobas 6000 (c501 module) in 2017 and 2018.

Other variables included age, sex, race, BMI, ratio of family income to poverty, education level, dietary intakes of energy and nutrients (protein, carbohydrate, and fat), prescription medication use, smoking status (whether smoked at least 100 cigarettes in life), heavy alcohol consumption (ever had 4/5 or more drinks every day), hypertension (mean systolic blood pressure ≥130 mmHg, mean diastolic blood pressure ≥80 mmHg, current use of antihypertensive medications, or self-reported physician-diagnosed hypertension) (19, 20), diabetes (self-reported physician-diagnosed diabetes or glycohemoglobin (HbA1c) ≥6.5% in those without a self-reported diagnosis) (21), weak/failing kidneys (self-reported physician-diagnosed weak/failing kidneys or estimated glomerular filtration rate (eGFR) <60 mL/min/1.73 m^2^) (22), vigorous work activity and laboratory variables (blood urea nitrogen, cholesterol, triglycerides). These data can be found on the NHANES website.^1^

### Statistical Analyses

The baseline characteristics were shown as means ± SDs (standard deviation) and percentages for continuous variables and categorical variables, respectively. In our study, obesity was defined as TPF >25% in males and >35% in females according to the American Association of Clinical Endocrinologists (AACE) and the American College of Endocrinology (ACE) (23). Multivariable linear regression models were used to estimate βs and their 95% CIs for the relationship between TPF and SUA. No covariate was adjusted in Model 1. Model 2 analysis was adjusted for age, sex, and race to account for potential demographic confounders. Model 3 analysis was additionally adjusted for health-related behaviors (smoking status, heavy alcohol consumption, and vigorous work activity), hypertension, diabetes, weak/failing kidneys, BMI, blood urea nitrogen, cholesterol, triglycerides, education level, ratio of family income to poverty, dietary intakes of energy and nutrients (protein, carbohydrate, and fat), and prescription medication use. Subgroup analyses stratified by sex and obesity status were also performed by multivariable regression. Furthermore, the non-linear relationship between TPF and SUA was addressed by smoothing and generalized additive models. When non-linearity was detected, a recursive algorithm was used to calculate the inflection point. Then, a two-segment piecewise linear regression model was used to analyze the relationship between TPF and SUA on both sides of the inflection point. All analyses were performed using R^2^ and EmpowerStats,^3^ *p*-values less than 0.05 were considered to indicate statistical significance.

## Results

The weighted characteristics of study samples based on obesity status are shown in Table 1. Overall, there were 15,808 (66.7%) obese individuals in all 23,715 participants. Compared with the non-obese group, the obese group showed older age, higher measurements of SUA, cholesterol, triglycerides, BMI, but lower dietary intakes of energy and nutrients (protein, carbohydrate, and fat). Additionally, there were higher proportions of Hispanics, females, hypertension, diabetes, prescription medication use, but lower proportion of vigorous work activity in the obese group compared with the non-obese group.

**TABLE 1:** Weighted characteristics of study samples based on obesity status.

	Non-obese (*n* = 7,907)	Obese (*n* = 15,808)	*p*-value
Total percent fat (%)	24.89 ± 5.89	36.71 ± 7.08	<0.01
Serum uric acid (μmol/L)	296.98 ± 75.70	325.33 ± 82.98	<0.01
Age (years)	34.41 ± 11.65	40.33 ± 11.51	<0.01
Blood urea nitrogen (mmol/L)	4.49 ± 1.56	4.47 ± 1.48	0.29
Cholesterol (mmol/L)	4.73 ± 0.97	5.16 ± 1.08	<0.01
Triglycerides (mmol/L)	1.24 ± 1.35	1.83 ± 1.73	<0.01
BMI (kg/m^2^)	22.99 ± 3.19	30.85 ± 6.28	<0.01
Ratio of family income to poverty	2.98 ± 1.69	3.00 ± 1.65	0.30
Energy intake (kcal)	2417.24 ± 1016.61	2171.35 ± 900.03	<0.01
Protein intake (g)	91.74 ± 44.16	83.42 ± 37.89	<0.01
Carbohydrate intake (g)	292.55 ± 134.04	259.98 ± 117.61	<0.01
Fat intake (g)	89.53 ± 44.87	83.92 ± 42.22	<0.01
Sex (%)			<0.01
Males	56.20	47.69	
Females	43.80	52.31	
Race (%)			<0.01
Hispanic	12.88	17.60	
Non-Hispanic White	66.68	64.93	
Non-Hispanic Black	12.44	10.64	
Others	8.00	6.83	
Education level (%)			0.93
Less than high school	15.79	15.84	
High school and above	84.21	84.16	
Smoking status (%)			0.09
Yes	45.42	44.22	
No	54.58	55.78	
Heavy alcohol consumption (%)			0.89
Yes	16.04	15.97	
No	83.96	84.03	
Hypertension (%)			<0.01
Yes	25.36	45.11	
No	74.64	54.89	
Diabetes (%)			<0.01
Yes	2.92	9.54	
No	97.08	90.46	
Weak/failing kidneys (%)			<0.01
Yes	2.68	2.12	
No	97.32	97.88	
Vigorous work activity (%)			<0.01
Yes	41.92	30.31	
No	58.08	69.69	
Prescription medication use (%)			<0.01
Yes	37.70	51.12	
No	62.30	48.88	

*Mean ± SD for continuous variables and p-value was calculated by weighted linear regression model.% for categorical variables and p-value was calculated by weighted chi-square test.*

In model 1 with no covariate adjusted and model 2 with age, sex, and race adjusted, TPF was positively associated with SUA (model 1: β = 3.38, 95% CI: 3.25–3.51; model 2: β = 3.55, 95% CI: 3.41–3.68). When fully adjusted for age, sex, race, health-related behaviors (smoking status, heavy alcohol consumption, vigorous work activity), hypertension, diabetes, weak/failing kidneys, BMI, blood urea nitrogen, cholesterol, triglycerides, education level, ratio of family income to poverty, dietary intakes of energy and nutrients, and prescription medication use, TPF was still positively associated with SUA (model 3: β = 0.99, 95% CI: 0.73–1.26), suggesting a 1% increase in TPF was accompanied by a 0.99 μmol/L increase in SUA. Additionally, subgroup analyses stratified by sex in these three models showed the same trend in both males and females (*Males*, model 1: β = 3.35, 95% CI: 3.15–3.56; model 2: β = 3.73, 95% CI: 3.52–3.93; model 3: β = 1.72, 95% CI: 1.32–2.12. *Females*, model 1: β = 3.40, 95% CI: 3.23–3.57; model 2: β = 3.40, 95% CI: 3.23–3.57; model 3: β = 0.38, 95% CI: 0.03–0.72). When stratified by obesity status, the positive association remained statistically significant in the adjusted models. The results of these models are presented in Table 2.

**TABLE 2:** The association between total percent fat (%) and serum uric acid (μmol/L).

	Model 1 β (95% CI) *p*-value	Model 2 β (95% CI) *p*-value	Model 3 β (95% CI) *p*-value
**Total**	3.38 (3.25, 3.51) < 0.0001	3.55 (3.41, 3.68) < 0.0001	0.99 (0.73, 1.26) < 0.0001
**Sex**			
Males	3.35 (3.15, 3.56) < 0.0001	3.73 (3.52, 3.93) < 0.0001	1.72 (1.32, 2.12) < 0.0001
Females	3.40 (3.23, 3.57) < 0.0001	3.40 (3.23, 3.57) < 0.0001	0.38 (0.03, 0.72) 0.03
**Obesity status**			
Non-obese	–5.45 (–5.71, –5.20) < 0.0001	2.97 (2.57, 3.38) < 0.0001	1.43 (0.84, 2.02) < 0.0001
Obese	–3.69 (–3.86, –3.51) < 0.0001	3.81 (3.56, 4.07) < 0.0001	0.62 (0.19, 1.05) 0.0051

*No covariate was adjusted in Model 1. Model 2 indicates that analysis was adjusted for age, sex, and race. Model 3 indicates model 2 adjustment plus the adjustment for health-related behaviors (smoking status, heavy alcohol consumption, vigorous work activity), hypertension, diabetes, weak/failing kidneys, body mass index, blood urea nitrogen, cholesterol, triglycerides, education level, ratio of family income to poverty, dietary intakes of energy and nutrients (protein, carbohydrate, and fat), and prescription medication use. Sex was not adjusted in the sex-stratified subgroup analyses.*

**FIGURE 1:**
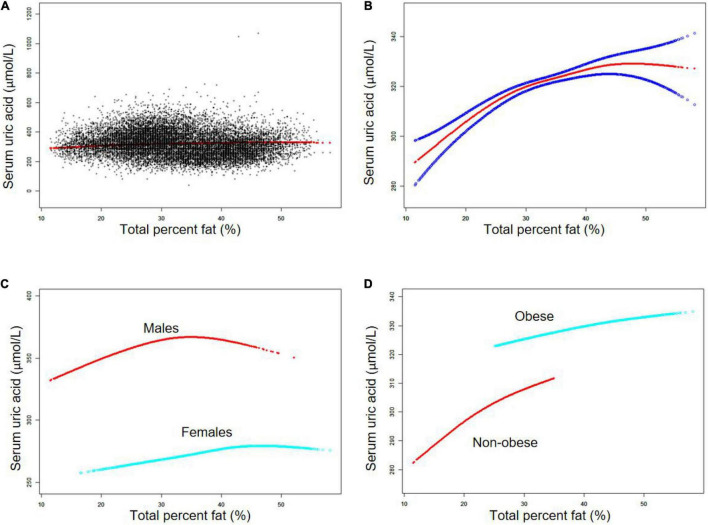
The association between total percent fat (%) and serum uric acid (μmol/L). **(A)** Each black point represents a sample. **(B)** Solid red line represents the smooth curve fit between variables. Blue bands represent the 95% confidence bands derived from the fit. **(C)** Stratified by sex. **(D)** Stratified by obesity status. Age, sex, race, smoking status, heavy alcohol consumption, vigorous work activity, hypertension, diabetes, weak/failing kidneys, body mass index, blood urea nitrogen, cholesterol, triglycerides, education level, ratio of family income to poverty, dietary intakes of energy and nutrients (protein, carbohydrate, and fat), and prescription medication use were adjusted (C was not sex-adjusted).

The non-linear relationship between TPF and SUA was further captured by smooth curve fittings and generalized additive models (Figures 1A,B). In the subgroup analysis, an inverted U-shaped relationship between TPF and SUA was observed in males (Figure 1C), with the inflection point at 34.3% TPF which was identified by a two-segment piecewise linear regression model. Moreover, in males with TPF < 34.3%, every 1% increase in TPF was accompanied by a 1.97 μmol/L increase in SUA (95% CI: 1.55–2.38). By contrast, in males with TPF > 34.3%, a 1% increase in TPF was accompanied by a 0.96 μmol/L decrease in SUA, but the *P*-value indicated no statistical significance (95% CI: -2.24 to 0.33). The results are shown in Table 3. The subgroup analysis stratified by obesity status is shown in Figure 1D.

**TABLE 3:** Threshold effect analysis of total percent fat (%) and serum uric acid (μmol/L) using the two-segment piecewise linear regression model.

	Males β (95% CI) *p*-value
**Fitting by standard linear model**	
	1.72 (1.32, 2.12) <0.0001
**Fitting by two-segment piecewise linear model**	
Inflection point	34.3
TPF < Inflection point	1.97 (1.55, 2.38) <0.0001
TPF > Inflection point	–0.96 (–2.24, 0.33) 0.1452
Log-likelihood ratio	<0.001

## Discussion

Our study demonstrated that TPF was positively and significantly related to SUA in adults aged 18–59 years in all three models analyzing the NHANES 1999–2018 data. Sex- and obesity-stratified analyses revealed that TPF was still positively and significantly associated with SUA in the adjusted models. Additionally, the inverted U-shaped non-linear relationship between TPF and SUA was identified in males with the inflection point at 34.3% TPF.

In the past few decades, several epidemiological studies have shown the positive association between obesity and SUA. However, the majority of them used the BMI approach to measure obesity, and very few of them focused on the association between TPF and SUA. For example, Chen et al. (14), Dai et al. (24), and Tanaka et al. (25), all observed the strong positive correlation between BMI and SUA in different groups of individuals. However, the primary concern of the BMI approach was that it could not identify normal weight obesity, which is defined as the combination of high percentage of body fat and normal BMI (26). Thus, more assessment methods of obesity were developed to explore the relationship between obesity and SUA. In early 1997, Takahashi et al. showed that in a cohort of 50 healthy males, the accumulation of visceral fat measured by abdominal computed tomography (CT) at the level of the umbilicus may have a greater adverse effect on UA metabolism than BMI (27). In 1998, Matsuura et al. investigated the relationship between body fat distribution measured by CT and UA metabolism in 36 obese males. They found that the frequency of hyperuricemia (HUA) was markedly higher in the visceral fat obesity group compared with the subcutaneous fat obesity group, suggesting the crucial role of fat distribution pattern in UA metabolism (28). Subsequently, some large-scale cross-sectional studies were carried out to investigate this relationship. For instance, a study involving 508 males found a close relationship between the increased CT-measured visceral adiposity and the high risk of HUA after adjusting for BMI, blood pressure and other confounders (29). Additionally, a cohort containing 699 individuals with diabetes showed the same findings and it further found a 2.33-fold increased risk of HUA in subjects with high visceral adiposity (30). A recent study showed that in patients with type 2 diabetes, the waist circumference (WC) and visceral adipose tissue (VAT) mass were independent risk factors for HUA (16). It was also shown that the adipokines released from VAT had potential effects on SUA production (31), and the reduction of VAT may contribute to the significant decline in SUA (32).

Furthermore, not only was obesity associated with a higher risk of HUA, an elevated SUA was also proven to be independently linked to the development of obesity (17, 33). Studies have shown that SUA can increase fat storage, elevate triglyceride levels (34), and predict subsequent weight gain in humans (33, 35). An *in vivo* study further showed that an SUA-lowering drug (allopurinol) can both prevent HUA and weight gain in rats (36). Besides, HUA was also suspected to cause obesity through accelerating hepatic and peripheral lipogenesis (37). In addition, lowering SUA can prevent fructose-induced fat accumulation in HepG2 cells and reduce hepatic steatosis in a mouse model of metabolic syndrome (38). Recently, a cross-sectional study demonstrated a strong association between SUA and VAT (measured by magnetic resonance imaging) after adjustment for BMI or WC (39). We also noticed that in a study of 7,544 participants ≥ 40 years from the NHANES III, the highest SUA group (>8 mg/dL) participants were twice as likely to show sarcopenia compared to the lowest SUA group (<6 mg/dL) participants (40). Moreover, there seemed to be a vicious circle between SUA and fat mass accumulation since the secretion of SUA from adipose tissue may be enhanced in obese individuals (41, 42). Additionally, a strong association with metabolic syndrome was also found in children and adolescents with high adiposity index or high SUA (43, 44).

Several pathophysiological and metabolic mechanisms have been proposed to explain the abovementioned association between adipose tissue and SUA. On the one hand, UA secreted from the adipose tissue was enhanced through the increased production of xanthine oxidoreductase (XOR) in obese individuals (45). Additionally, the obesity-linked insulin resistance can also inhibit the excretion and promote the reabsorption of SUA (46, 47). On the other hand, SUA may also induce insulin resistance via inhibiting the availability of nitric oxide and decreasing insulin-mediated glucose uptake in skeletal muscles (48, 49), and the elevated SUA may damage islet B cells directly to aggravate insulin resistance (50). In addition, the accumulated insulin can inhibit the decomposition of visceral fat, leading to the development of obesity (51). And it was also indicated that SUA might take part in stimulating the secretion of inflammatory markers such as C-reactive protein (CRP), interleukin (IL)-6, IL-18, and tumor necrosis factor (TNF)-alpha which are crucial to the development of metabolic syndrome (52, 53).

Interestingly, an earlier study showed that estrogen can lower SUA in postmenopausal women (54) and trans-sexual men (55), which can be explained by the possible enhancement of SUA excretion by estrogen (56). Specifically, estrogen was found to decrease urate reabsorptive transporter expression at the posttranscriptional level, resulting in increased UA excretion and decreased SUA levels (57). Thus, estrogen may account for the different patterns of association between TPF and SUA in different sexes, but the detailed mechanisms still need to be further investigated. In our study, the non-linear relationships between TPF and SUA differed in males and females. With the increase of TPF, a steady increase in SUA was observed in females, while an inflection point was identified in males. Moreover, other studies showed that the relationship between SUA and the incidence of coronary heart disease, hypertension and renal dysfunction was stronger in females than in males (58–60), suggesting that females might be more vulnerable to organ damage caused by UA, which was consistent with the observation that females had lower UA levels than males (61). Thus, it would be desirable to take into account sex-related differences while implementing measures to prevent HUA caused by obesity.

There are some strengths and limitations of our study. First, it was a large-scale study involving nationally representative American adults with complete TPF and SUA data (*n* = 23,715), which made it possible for us to conduct subgroup analysis to further illustrate the different patterns of association between TPF and SUA in males and females. Second, the TPF data in our study were measured by DXA, which offered a more accurate measurement of adipose tissue compared with the traditional BMI-based approach. The limitations of this study are also worthy of note. First and foremost, it was the nature of cross-sectional study that the outcomes and exposures were ascertained at the same time that limited the inference of a causal relationship between TPF and SUA. Thus, large-sample prospective studies are further needed to explore this causality. Second, the TPF data of participants ineligible for DXA scan (e.g., excess body weight or height) were not included in the original survey. Thus, the selection bias cannot be avoided. Third, some covariates in our study such as hypertension and diabetes status, smoking and drinking status, as well as physical activity level were all based on self-report data, which may introduce the recall bias. Finally, little was known about the potential biases caused by other unrecognized confounding factors, leaving them unadjusted in this study.

## Conclusion

In conclusion, our results supported a significant positive association between TPF and SUA, even after the adjustment for a wide range of potential confounders. And this association remained statistically significant when stratified by sex and obesity status. However, the underlying mechanisms of the different patterns of this relationship in males and females still need further investigation.

## Data Availability Statement

Publicly available datasets were analyzed in this study. These data can be found here: http://www.cdc.gov/nchs/nhanes.

## Author Contributions

JS and CY contributed to data collection, statistical analysis, and writing of the manuscript. ZL and JL contributed to data collection and statistical analysis. WK supervised the study and contributed to polishing and reviewing of the manuscript. All authors contributed to the article and approved the submitted version.

## Conflict of Interest

The authors declare that the research was conducted in the absence of any commercial or financial relationships that could be construed as a potential conflict of interest.

## Publisher’s Note

All claims expressed in this article are solely those of the authors and do not necessarily represent those of their affiliated organizations, or those of the publisher, the editors and the reviewers. Any product that may be evaluated in this article, or claim that may be made by its manufacturer, is not guaranteed or endorsed by the publisher.

## Footnotes

1
http://www.cdc.gov/nchs/nhanes/

2
http://www.Rproject.org

3
http://www.empowerstats.com
